# Draft Genome Sequences of Plant-Pathogenic Klebsiella variicola Strains Isolated from Plantain in Haiti

**DOI:** 10.1128/MRA.00336-20

**Published:** 2020-07-16

**Authors:** James C. Fulton, Jeannie M. Klein, Sladana Bec, Joubert Fayette, Karen A. Garrett, Jeffrey B. Jones, Sujan Timilsina, Carrie L. Harmon

**Affiliations:** a Department of Plant Pathology, University of Florida, Gainesville, Florida, USA; b Institute for Sustainable Food Systems, University of Florida, Gainesville, Florida, USA; University of Arizona

## Abstract

The genus *Klebsiella* includes pathogenic and nonpathogenic species. We report the 5.57-Mb genome sequences of two Klebsiella variicola strains, G18-1365 and G18-1376, isolated from symptomatic plantain plants in Haiti. These strains are genetically closely related (average nucleotide identity [ANI] > 99%) to the previously described type strain of *K. variicola*, DSM 15968.

## ANNOUNCEMENT

A member of the *Enterobacteriaceae* family, the Gram-negative genus *Klebsiella* includes species found globally in a wide range of habitats, some of which are important animal and plant pathogens (1–3). Using strains isolated from plant and nosocomial patient samples, *K. variicola* was first classified as a distinct species in 2004 based on DNA-DNA hybridization and multilocus sequence analysis (MLSA) of six housekeeping genes (4). We previously reported isolation of *K. variicola* strains from symptomatic plantain plants exhibiting necrotic soft rot in several Haitian farms (5). The bacteria were preliminarily identified as *K. variicola* using MLSA of the rRNA, *rpoB*, *phoE*, and *infB* genes and phenotype microarray profiles (Biolog, Hayward, CA). To confirm that the strains were most closely related to *K. variicola*, a subset of strains was subjected to whole-genome sequencing.

Single colonies previously preserved in 20% glycerol and stored at −80°C were grown for 24 h at 28°C on nutrient agar (BBL; Becton, Dickinson and Co., Franklin Lakes, NJ), and genomic DNA was extracted using a Wizard genomic DNA purification kit (Promega, Chicago, IL). Library preparation and genome sequencing were performed at the Microbial Genome Sequencing Center (Pittsburgh, PA). Tagmentation, as described by Baym et al. (6), was used to prepare the genomic libraries. A NextSeq 550 system (Illumina, San Diego, CA) was used to produce 151-bp paired-end reads. Trim Galore! v. 0.6.3 with default parameters was used to trim and pair the raw reads, which were then assembled into contigs with SPAdes v. 3.10.1 with k-mers 21, 33, 55, 77, 99, and 127. Contigs smaller than 500 bp and with less than 2.0 k-mer coverage were removed. Bowtie 2 v. 2.3.3 was used with default parameters to align the validated reads against the filtered contigs and output a sequence alignment map (SAM)-formatted alignment. SAMtools was used to convert the SAM files to binary alignment map (BAM) files; then, Pilon v. 1.22 was used with default parameters to polish the draft assemblies and output improved FASTA files.

Assembled sequences were annotated using the Department of Energy Joint Genome Institute’s Integrated Microbial Genomes and Microbiomes annotation pipeline v. 5.0.3 (7, 8) and the National Center for Biotechnology Information’s internal Prokaryotic Genome Annotation Pipeline (9). The final assembly for G18-1365 was 5,572,087 bp long with a GC content of 57.35%, 43 contigs, and 87.03× coverage. The annotation for strain G18-1365 (IMG Genome ID 28356457) predicted 5,235 protein-coding genes and 173 RNA genes. The final assembly for G18-1376 was 5,572,027 bp long with a 57.35% GC content, 41 contigs, and 86.92× coverage. The annotation for strain G18-1376 (IMG Genome ID 2835651175) predicted 5,229 protein-coding genes and 178 RNA genes. Average nucleotide identity comparisons between newly sequenced strains G18-1365 and G18-1376 and K. variicola strains At-22 (10), DSM 15968 (11), and 342 (12) were computed using methods described by Jain et al. (13), and the results are shown in Table 1.

**TABLE 1:** ANI^*a*^ comparisons of sequenced Klebsiella variicola genomes with representative publicly available Klebsiella variicola assemblies using FastANI calculations

Strain	ANI (%) for strain:		
	At-22	DSM 15968	342
G18-1365	99.07	99.06	98.94
G18-1376	99.07	99.06	98.93

a ANI, average nucleotide identity.

### Data availability.

These whole-genome sequences were deposited in GenBank with the raw data accession numbers PRJNA589638 and PRJNA589639 and assembly accession numbers GCA_009729475.1 and GCA_009729495.1 for G18-1365 and G18-1376, respectively.
